# Letter from the Editor in Chief

**DOI:** 10.19102/icrm.2024.150110

**Published:** 2024-01-15

**Authors:** Moussa Mansour


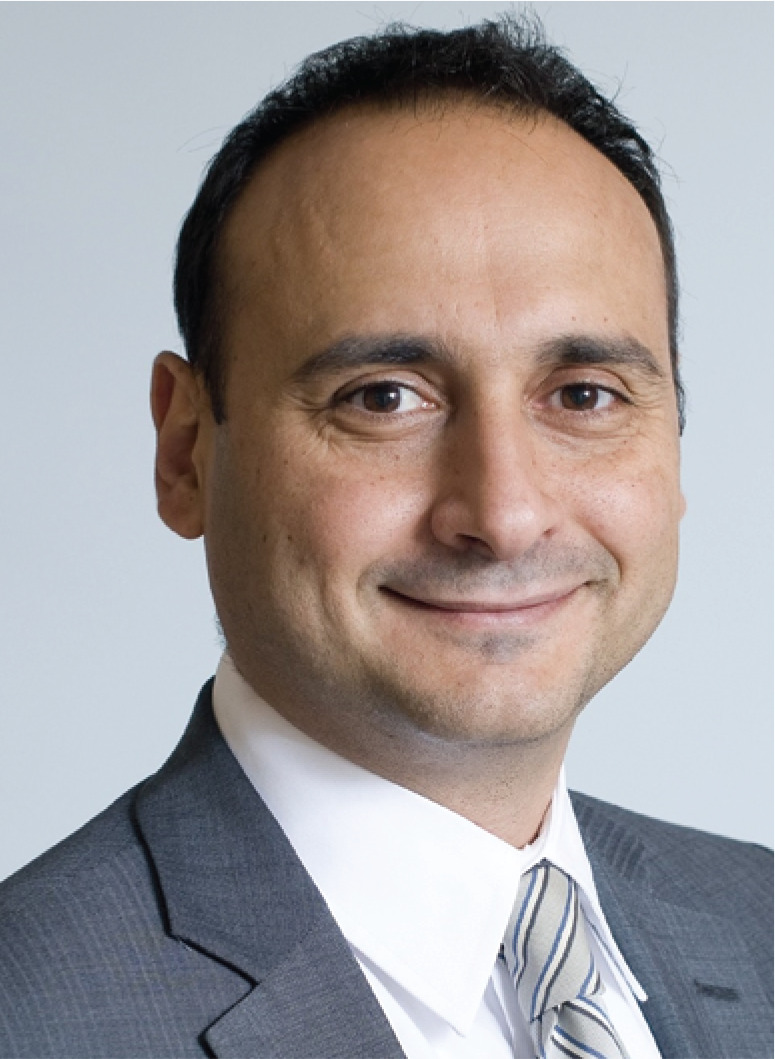


Dear readers,

The American College of Cardiology (ACC)/American Heart Association (AHA)/American College of Clinical Pharmacy/Heart Rhythm Society (HRS) guidelines for the diagnosis and management of atrial fibrillation (AF) were recently published,^1^ and the document has since been elegantly summarized by Wiggins et al.^2^ These recent guidelines contain significant updates compared to the prior version of the AHA/ACC/HRS guidelines^3^ and the focused update^4^ published in 2014 and 2019, respectively. Among the updates, I will review two that I believe will have the most impact on the clinical practice of interventional cardiac electrophysiology.

First is the increased emphasis on rhythm control. In addition to recommending rhythm control for symptomatic patients (Class of Recommendation IIa), the guideline provides recommendations for rhythm control in some patients regardless of the presence of symptoms. These patients include those with reduced ejection fraction (Class I) and those diagnosed with AF within 1 year of presentation (Class IIa). Rhythm control is also recommended to prevent the progression of AF from paroxysmal to persistent (Class IIb) and to reduce the likelihood of dementia onset (Class IIb). More specifically, rhythm control to reduce symptoms using catheter ablation as a first-line therapy received a Class I recommendation for young patients with paroxysmal AF and few comorbidities, and a Class IIa recommendation for a less-restrictive patient population, including those with paroxysmal and persistent AF, with more comorbidities.

The second update of note is the upgraded recommendation for percutaneous left atrial appendage (LAA) occlusion (LAAO), which received a Class IIa recommendation for patients with AF, a moderate to high risk of stroke (CHA_2_DS_2_-VASc score ≥ 2 points), and a contraindication to long-term oral anticoagulation (OAC). Contraindication to OAC was well defined in the document and included severe bleeding due to a non-reversible cause, spontaneous intracranial/intraspinal bleeding due to a non-reversible cause, and serious bleeding related to recurrent falls when the cause of falls was not felt to be treatable. This differs from the 2019 version, where LAAO had a Class IIb recommendation for the same clinical scenario. Moreover, in the current 2023 version, LAAO as an alternative to OAC received a Class IIb recommendation for patients who are at high risk for bleeding, based on patient preference, with careful consideration of procedural risk and with the understanding that the evidence for oral anticoagulation is more extensive.

In summary, the latest guidelines provided upgraded recommendations for two cardiac EP procedures: catheter ablation and LAA closure. This was the result of landmark well-designed clinical studies that demonstrated the efficacy and safety of these two procedures, and these changes are expected to enhance clinical practice going forward.

I hope that you enjoy reading this issue of *The Journal of Innovations in Cardiac Rhythm Management*.


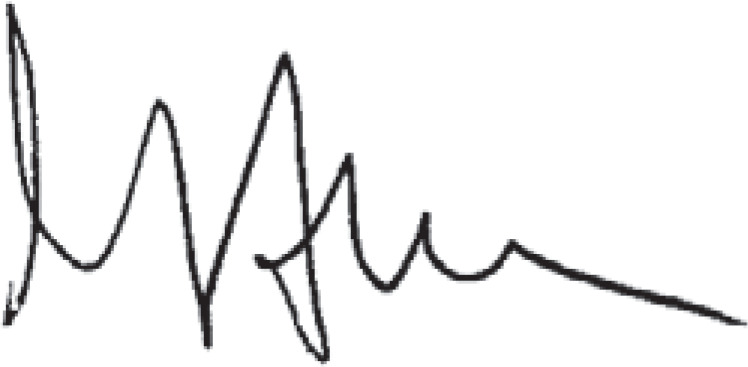


Sincerely,

Moussa Mansour, md, fhrs, facc

Editor in Chief

*The Journal of Innovations in Cardiac Rhythm Management*

MMansour@InnovationsInCRM.com

Director, Atrial Fibrillation Program

Jeremy Ruskin and Dan Starks Endowed Chair in Cardiology

Massachusetts General Hospital

Boston, MA 02114
